# MicroRNA-485 plays tumour-suppressive roles in colorectal cancer by directly targeting GAB2

**DOI:** 10.3892/or.2022.8274

**Published:** 2022-01-27

**Authors:** Jibin Li, Jian Xu, Xiaofei Yan, Keer Jin, Wenya Li, Rui Zhang

Oncol Rep 40: 554-564, 2018; DOI: 10.3892/or.2018.6449

Following the publication of this paper, the authors contacted the Editorial Office to request that the article be retracted on account of an inability to obtain consistent results from repeated experiments. Independently, it was drawn to the Editors attention that certain of the flow cytometric data shown in Figs. 2D, 5D and 6D were strikingly similar to data appearing in different form in another article by different authors.

Owing to the fact that the contentious data in the above article were already under consideration for publication prior to its submission to *Oncology Reports*, the Editor has agreed to the authors request that this article should be retracted from the Journal. The Editor apologizes to the readership for any inconvenience caused.

